# Malformation anévrismale de la veine de galien à Bamako à propos d’un cas

**DOI:** 10.11604/pamj.2019.34.52.19964

**Published:** 2019-09-26

**Authors:** Amadou Doumbia, Youssouf Kone

**Affiliations:** 1 Service d’Imagerie Médicale du Centre de Santé de Référence de la Commune VI, Bamako, Mali; 2 Service de Radiologie du Centre Hospitalier Jacques Boutard, Saint-Yrieix-la-Perche, France

**Keywords:** Malformation, veine de galien, anévrisme, Malformation, galen's vein, aneurysm

## Abstract

Galen aneurysmal malformation is a rare complex congenital vascular malformation of the brain, (less than 1% of intracranial arteriovenous malformations) characterized by pseudoaneurismal dilation of Galen's ampulla associated with one or several arteriovenous fistulas. In most cases, diagnosis is made in the neonatal or postnatal period, exceptionally in adulthood. We report the case of an 18-year male patient hospitalized for psychomotor retardation. Brain computed tomography (CT) scan using spontaneous contrast (A,B,C) showed voluminous isodense mass in the pineal region behind the third ventricle with peripheral calcifications, compatible with large vein of galen aneurysm. Its anteroposterior diameter was 8.5cm, its transverse diameter was 3cm in the axial plane (C) and its height was 3cm in the sagittal plane (G). Imaging objectified homogeneous contrast enhancement (D,E,F,G,H,I). The mass was associated with dilation of the superior sagittal sinus measured at 2.7cm (A,D), of transverse sinuses measured at 1.5cm to the right and at 1,1cm to the left (B,E). Multiple venous perianeurysmal derivations (I), hydrocephaly and small parenchymal cortical-subcortical calcifications were detected. Embolization can't be performed in Bamako, so the patient is waiting for transfer.

## Image en médecine

L'anévrisme de la veine de Galien est une malformation vasculaire cérébrale congénitale, rare (moins de 1% des malformations artério-veineuses intracrâniennes), complexe, réalisant une dilatation pseudo-anévrismale de l'ampoule de Galien associée à une ou plusieurs fistules artério-veineuses. Le diagnostic est néo ou post-natale dans la majorité des cas mais exceptionnel à l'âge adulte. Nous rapportons le cas d'un patient, âgé de 18 ans, de sexe masculin admis pour retard psychomoteur. La tomodensitométrie cérébrale en contraste spontané (A,B,C) objectivait une volumineuse masse isodense de la région pinéale en arrière du troisième ventricule avec des calcifications périphériques compatible avec la dilatation anévrysmale de la grande veine de Galien. Elle mesurait 8,5cm de diamètre antéropostérieur, 3cm de diamètre transverse en axiale (C) et 3cm de hauteur en sagittale (G). Il existait un rehaussement intense homogène (D,E,F,G,H,I). Elle était associée à une dilatation du sinus sagittal supérieur mesurée à 2,7cm (A,D), des sinus transverses mesurés à 1,5cm à droit et 1,1cm à gauche (B,E). Il existait également de multiples dérivations veineuses péri-anévrysmale (I), une hydrocéphalie et de petites calcifications parenchymateuses cortico-sous corticales. L'embolisation n'étant pas possible à Bamako, le patient est en attente d'une éventuelle évacuation sanitaire.

**Figure 1:**
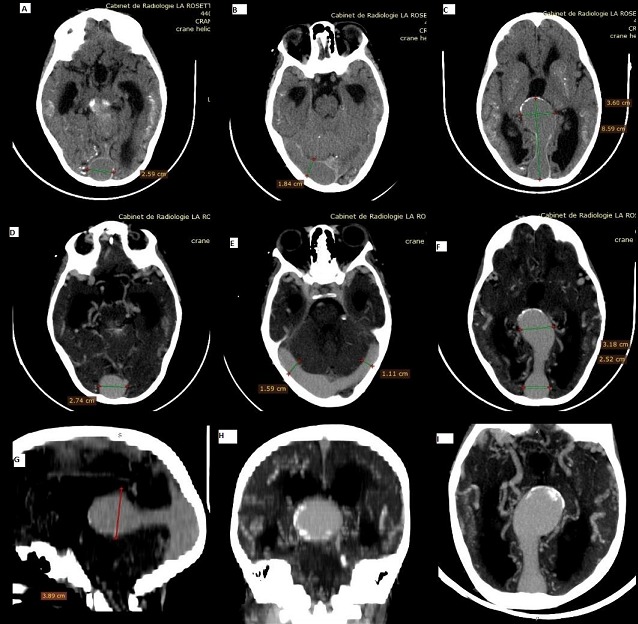
TDM cérébrale en contraste spontané (A,B,C): volumineuse masse isodense de la région pinéale, en arrière du V3 présentant des calcifications périphériques, mesurant 8,5cm de diamètre antéropostérieur, 3cm de diamètre transverse en axiale (C) et 3cm de hauteur en sagittale (G). Rehaussement intense homogène après injection (D,E,F,G,H,I). Dilatation du sinus sagittal supérieur à 2,7cm (A,D), des sinus transverses à 1,5cm à droit et 1,1cm à gauche (B,E). Multiples dérivations veineuses tortueuses péri-anévrysmale (I), Hydrocéphalie et de petites calcifications parenchymateuses cortico-sous corticale (A,B,C)

